# Frequency of metabolic syndrome in obesity: a comparison of current criteria of IDF and NCEP-ATPIII

**DOI:** 10.1186/1758-5996-7-S1-A148

**Published:** 2015-11-11

**Authors:** Luana Costa Nascimento, Mayne Batista Fontes Santos, Alexandre Rocha Andrade, Josilda Ferreira Cruz, Ana Denise Costa de Oliveira, Karla Freire Rezende, Rubens Cruz Silva Filho

**Affiliations:** 1 Universidade Federal de Sergipe, Aracaju, Brazil

## Background

Obesity appears as a worldwide epidemic, causing concerns and burden to public health. A very damaging complication of obesity is its relationship with the increase of the cardiovascular risk. We analyzed the association between obesity and the diagnosis of metabolic syndrome (MS) by following the two criteria in effect: NCEP-ATPIII and IDF.

## Objective

The aim of this work is evaluate the frequency of MS in patients with a previous diagnosis of obesity.

## Materials and methods

The study was a cross-sectional retrospective with 100 patients diagnosed with obesity.

## Results

The sample comprises 100 patients, 89 women and 11 men, with means of age 44.05 (±11.92) yrs. old, of weight 116.404 (±23.42) kg, of BMI 45.93 (±7.93) kg/m2 and of Abdominal Circumference of 129.08 (±15.531) cm. From the 100 patients, 34(%) reported performing pretreatment for DM2, 73(%) for hypertension and 48(%) for dyslipidemia. According to the NCEP-ATPIII, 73 patients had MS, from which 79.45% carried Morbid Obesity (MO) and 20.54% did not (p=0.564). According to the IDF, 88 patients had MS, from which 77.27% carried MO and 22.73% did not (p=0.634). The average age of MS patients was 45.66 (±11.89) and of non-carriers was 39.70 (±11.06) yrs. old (p=0.026), according to NCEP-ATPIII. According to IDF, the average age of MS patients was 44.94 (±12.12) and of non-carriers was 37.50 (±7.94) yrs. old (p=0.042). The average age of the MO was 42.85 (±11.77) and of the non-morbid was 48.32 (±11.72) yrs. old (p=0.057).

## Conclusion

There is an elevated frequency of MS diagnoses in obesity carriers, with the IDF criteria selecting more patients. It has also been demonstrated the high frequency of comorbidities among obese patients, being hypertension the most frequent of them. The average age was significantly higher at MS patients diagnosed by both criteria. There was also a tendency of patients carrying MO to be of a younger age. Despite the high frequency of MS in the studied patients, the progression of obesity degree does not lead to an increase of the SM frequency. There is an association between the average age and the presence of MS, being more frequent in older patients, but curiously not occurring with the BMI, which proved to be more biased in younger patients. This emphasizes the age as a possible contributing factor to the development of MS.

